# Lower expression of activating transcription factors 3 and 4 correlates with shorter progression-free survival in multiple myeloma patients receiving bortezomib plus dexamethasone therapy

**DOI:** 10.1038/bcj.2015.98

**Published:** 2015-12-04

**Authors:** T Narita, M Ri, A Masaki, F Mori, A Ito, S Kusumoto, T Ishida, H Komatsu, S Iida

**Affiliations:** 1 Department of Hematology and Oncology, Nagoya City University Graduate School of Medical Sciences, Nagoya, Aichi, Japan

## Abstract

Bortezomib (BTZ), a proteasome inhibitor, is widely used in the treatment of multiple myeloma (MM), but a fraction of patients respond poorly to this agent. To identify factors predicting the duration of progression-free survival (PFS) of MM patients on BTZ treatment, the expression of proteasome and endoplasmic reticulum (ER) stress-related genes was quantified in primary samples from patients receiving a combination of BTZ and dexamethasone (BD). Fifty-six MM patients were stratified into a group with PFS<6 months (*n*=33) and a second group with PFS⩾6 months (*n*=23). Of the 15 genes analyzed, the expression of activating transcription factor 3 (*ATF3*) and *ATF4* was significantly lower in patients with shorter PFS (*P*=0.0157 and *P*=0.0085, respectively). Chromatin immunoprecipitation analysis showed that these ATFs bind each other and transactivate genes encoding the pro-apoptotic transcription factors, *CHOP* and *Noxa*, which promote ER stress-associated apoptosis. When either ATF3 or ATF4 expression was silenced, MM cells partially lost sensitivity to BTZ treatment. This was accompanied by lower levels of Noxa, CHOP and DR5. Thus low basal expression of *ATF3* and *ATF4* may attenuate BTZ-induced apoptosis. Hence, ATF3 and ATF4 could potentially be used as biomarkers to predict efficacy of BD therapy in patients with MM.

## Introduction

Among novel agents targeting multiple myeloma (MM), the proteasome inhibitor, bortezomib (BTZ), was the first to be approved for the treatment of newly diagnosed MM in both transplant-eligible and non-eligible patients in Japan. It is considered a key drug for achieving prompt and meaningful responses. This agent strongly inhibits proteasome activity, which results in the disruption of homeostasis between protein synthesis and destruction.^1, 2^ BTZ treatment often results in excellent responses (partial response (PR) and complete response) not only in newly diagnosed MM but also in patients who have relapsed or are refractory to other treatments.^3^ Accordingly, it has significantly improved the prognosis of MM.^4^ However, not all patients treated with this agent experience such a favorable outcome. Suboptimal responses or lack of any response to BTZ is seen in a fraction of patients, and the efficacy of the agent is unpredictable. To date, few potential biomarkers positively associated with efficacy of BTZ treatment have been proposed.

It is well known that malignant tumor cells have abundant proteasome activity compared with normal cells. The purpose of this increased activity is probably to maintain proliferation and survival in the presence of apoptotic substrates.^5^ When the proteasome is inhibited, ubiquitinated proteins are not degraded and accumulate in the endoplasmic reticulum (ER). This can lead to ER stress and induce the unfolded protein response (UPR), occurring initially at the ER transmembrane.^6^ This response requires three activated ER transmembrane proteins, namely, PKR-like ER kinase (PERK), activating transcription factor 6 (ATF6) and inositol-requiring kinase 1 (IRE1α).^7, 8^ Activation of these stress sensor proteins results in the transcriptional activation of various UPR target genes, including ER-resident chaperones, ER-associated degradation (ERAD) components and pro-apoptotic factors. When the extent of ER stress is limited, the UPR mainly acts to neutralize its effects through three compensatory mechanisms, namely, the reduction of new protein synthesis to avoid a severe burden on the ER, repair of unfolded proteins with the aid of ER chaperones and exclusion of misfolded proteins from the ER to be degraded by the proteasome. Of the three ER transmembrane proteins, phosphorylated PERK adjusts the translation of new proteins and upregulates transcription factor ATF4 followed by further production of ER chaperones. ATF6 is cleaved at the ER transmembrane when misfolded protein accumulates, and the cytosolic portion of the substrate moves to the nucleus and acts as a transcription factor to promote transcription of ER chaperones. Activated IRE1α possesses two functional enzymatic domains, an autophosphorylation kinase and an endonuclease kinase domain, by which it oligomerizes and carries out unconventional RNA splicing. This results in an intron being removed from the X-box-binding protein 1 (XBP1) mRNA.^9^ Spliced XBP1 (XBP1s) is thus freed to become a functional transcription factor and upregulates ER chaperones and ERAD genes that facilitate recovery from ER stress.^9, 10^ However, when cellular stress is too great for these compensatory mechanisms, the UPR changes from acting to promote cellular survival to committing the cell to apoptosis through upregulation of pro-apoptotic transcription factors. Among several cellular stresses, proteasome inhibition can lead to ER stress that cannot be compensated for, resulting in upregulation of ATF4 followed by ATF3 expression. Heterodimerization of these substrates then promotes cell death, with enhancement of pro-apoptotic factors.^11, 12, 13, 14^

From previous studies, ER stress and subsequent UPR are recognized as the main mechanisms of BTZ-induced apoptosis.^15, 16, 17^ In addition, several studies^18, 19^ have reported associations of expression levels of genes in the IRE1-XBP1 pathway with BTZ sensitivity, based on the analysis of patients with MM receiving BTZ-containing therapy, and have suggested that low expression of *XBP1* in primary MM cells is associated with a poor response to BTZ-containing therapy or poor prognosis. Therefore, it is possible that evaluation of expression of these genes may predict the efficacy of BTZ treatment in MM. To test this hypothesis, we assessed basal expression levels of proteasome and ER stress-related genes in primary myeloma samples from patients receiving BTZ and dexamethasone (DEX) (BD) combination therapy, which mainly consisted of intravenous or subcutaneous administration of BTZ and oral administration of low-dose DEX. We evaluated the relationship between the level of expression of each gene and treatment efficacy parameters. Among such genes, we found that two ATF substrates, ATF3 and ATF4, are expressed at lower levels in poor responders to BD. Low basal expression of *ATF* genes was associated with prevention of BTZ-induced apoptosis through suppression of the induction of the pro-apoptotic factors CHOP and Noxa at the transcriptional level.

## Materials and methods

### Isolation of primary MM specimens and subsequent experimental conditions

Fifty-six primary specimens from patients with MM were collected prior to BD treatment after written informed consent was obtained at Nagoya City University Hospital. The assay protocols using patient samples were approved by the Institutional Ethical Committee. Primary MM cells were isolated from the bone marrow (BM) mononuclear cell fraction using anti-CD138 antibody-coated beads with the aid of an automatic magnetic cell sorting system (Miltenyi Biotec, Auburn, CA, USA).^17^ To minimize the effect of contamination with normal plasma cells, only those BM specimens for which clonal proliferation of MM cells had been confirmed by both pathological diagnosis and flow cytometric analysis were selected for use in this study. In addition, to standardize the experimental conditions and minimize the stress in tumor cells that occurs under *in vitro* condition, all BM specimens were selected immediately after harvesting, stored uniformly within 1 h and RNA was extracted without delay from all primary MM samples.

### Cell culture and reagents

Two human MM cell lines, KMS-11 and RPMI-8226, were cultured as described previously.^10^ BTZ was purchased from Toronto Research Chemicals (North York, Ontario, Canada). Antisera against ATF3, CHOP and actin were purchased from Santa Cruz Biotechnology (Santa Cruz, CA, USA). Antisera against ATF4, DR5 and cleaved caspase 3 were purchased from Cell Signaling Technology (Danvers, MA, USA). Antiserum against Noxa was purchased from Merck4 Biosciences (Darmstadt, Germany).

### Apoptosis assays

Apoptosis of cells exposed to BTZ for 72 h was evaluated using propidium iodide (PI; Sigma-Aldrich, St Louis, MO, USA). The fraction of PI-positive cells was determined using a FACS Calibur (BD Biosciences, San Jose, CA, USA).

### Western blotting analysis

MM cell lines and primary tumor cells from patients with MM were incubated with or without BTZ for 12–24 h. Preparation of whole-cell extracts and their analysis was carried out as described previously.^13^ Each loaded sample was adjusted to 30 μg per 10 μl, after estimating protein content using Bradford reagents.

### Quantitative real-time reverse transcription-PCR

Total RNA was extracted from purified MM cells using the RNeasy Mini Kits (Qiagen, Valencia, CA, USA). Reverse transcription and amplification of total RNA was performed using the CellAmp Whole Transcriptome Amplification Kit (Takara Bio, Shiga, Japan). This kit is designed to generate cDNA derived from mRNA using oligo dT-primers and facilitate uniform cDNA amplification by PCR when real-time PCR primers correspond to a position within 1 kb of the 3′ end of mRNA transcripts. Quantitative PCR was carried out using SYBR Green Gene Expression Assays (Toyobo, Osaka, Japan) and a Step One Plus Real-Time PCR instrument (Applied Biosystems, Foster City, CA, USA) according to the manufacturer's instructions. Twelve primer sets, PSMB5, PSMB6, PSMB7, PSMB8, PSMB9, EIF2AK3, EIF2S1, ERN1, XBP1, ATF3, ATF5 and DDIT3, were purchased from Takara Bio. Three primers were designed as follows: for PSMB10 5′-CGGTCGTGGCGGACAA-3′ and 5′-GCCCCACAGCAGTAGATTTTG-3′ for ATF6 5′-ACGGAGTATTTTGTCCGCCT-3′ and 5′-TGCAGCTCATCAGTGTCTGT-3′ and for ATF4 5′-TCCGAATGGCTGGCTGTGG-3′ and 5′-AGTGTAGTCTGGCTTCCTATCTCC-3′. The values of all samples are from the means of two determinations, calculated from relative standard curves originating from amplified cDNA of the KMS-11 cell line and finally adjusted to the expression of β-glucuronidase mRNA as an endogenous control.

### Stable knockdown of ATF3 and ATF4 expression by lentiviral microRNA (miRNA)

miRNA targeting ATF3 or ATF4 was chemically synthesized, annealed, terminally phosphorylated and inserted into an entry vector using BLOCK-iT Pol II miR RNAi Expression Vector Kits (Invitrogen, Carlsbad, CA, USA). The sequences of miRNA were as follows: for ATF3 top strand 5′-TGCTGAATCCTCAAACACCAGTGACCGTTTTGGCCACTGACTGACGGTCACTGGTTTGAGGATT-3′ and antisense 5′-CTGAATCCTCAAACCAGTGACCGTCAGTCAGTGGCCAAAACGGTCACTGGTGTTTGAGGATTC-3′ and for ATF4 sense strand 5′-TGCTGTCTATGTACAAGCACATTGACGTTTTGGCCACTGACTGACGTCAATGTTTGTACATAGA-3′ and antisense 5′-CCTGTCTATGTACAAACATTGACGTCAGTCAGTGGCCAAAACGTCAATGTGCTTGTACATAGAC -3′. Ineffective sequences were used as controls, designated 'negative'. The lentivirus-based expression vector was constructed as a combination of the miRNA-containing entry vector, cytomegalovirus promoter-containing vector and plenti6.4/R4R2/V5-DEST multisite gateway vector using BP and LR reactions. Lentiviruses were produced and harvested as previously described.^17^ Cells from the KMS-11 cell line were infected with miRNA-containing lentivirus for 24 h, followed by incubation with 10 μg/μl blasticidin for 2 or 3 weeks. After incubation, stable clones were enriched by selecting green fluorescent protein-positive cells using a BD FACSAria 2 cell sorter (BD Biosciences). Knockdown efficiency was confirmed by western blotting.

### Chromatin immunoprecipitation (ChIP) assay

Assays were performed using the EpiQuick Chromatin Immunoprecipitation Kit (Epigentek, Farmingdale, NY, USA). MM cells treated with 10 nm BTZ were crosslinked by the addition of 37% formaldehyde to the medium at a final concentration of 1% and incubation for 60 min at room temperature. Crosslinking was stopped by addition of glycine to a final concentration of 0.125 m and incubation for 5 min at room temperature. Cells were washed with ice-cold phosphate-buffered saline, and cell pellets were resuspended in lysis buffer with protease inhibitors and incubated on ice. The chromatin was sheared by sonication, plated into microwells and immobilized with antibody targeting ATF3 or ATF4 antigen, using normal mouse immunoglobulin G as a control. In this technique, crosslinked DNA is released from antibody-captured protein–DNA complexes, reversed and purified through the specifically designed Fast-Spin Column. Eluted ChIP DNA was analyzed by PCR using four primer pairs for the promoter sequences of ATF3, ATF4, CHOP and Noxa. The sequences of primers were as follows: for ATF3 5′-GGACTGGCAACACGGAGTAA-3′ and 5′-GGCGAGAGAAGAGAGCTGTG-3′ for ATF4 5′-TAAACGGTTGGGGCGTCAAA-3′ and 5′-CGCCGGCCCTTTATAGACTT-3′ for DDIT3 5′-CATCCGCCACTCAGGAGC-3′ and 5′- TGAAGCCTCGTGACCCAAAG-3′ and for Noxa 5′-CCTACGTCACCAGGGAAGTT-3′ and 5′-GATGCTGGGATCGGGTGT-3′.

### Statistical analysis

Analyses were carried out using GraphPad Prism 5 software (GraphPad Software, San Diego, CA, USA). Comparison of gene expression between two groups with longer and shorter PFS after BD therapy was made using the Mann–Whitney *U*-test. Correlations between gene expression levels and PFS were established with Spearman's correlation coefficient by rank. Survival analysis used the Kaplan–Meier estimate. In this study, *P*<0.05 was considered significant.

## Results

### Characterization of MM study patients receiving BD therapy

Between May 2007 and March 2014, all MM patients receiving BD treatment in our institute were candidates for this study. Most had been previously treated and received BTZ weekly or twice weekly in 1.3 mg/m^2^ doses intravenously or subcutaneously. Oral or intravenous administration of 20 mg of DEX was given on the day of BTZ treatment and on the following day. Of these patients, 56 with BM specimens collected prior to initial BD therapy were chosen for analysis. As the time to event was more important than the degree of response to assess therapeutic efficacy, these patients were divided into two groups according to the duration of progression-free survival (PFS) <6 months (*n*=33) or ⩾6 months (*n*=23). The results of several previous studies on relapsed or refractory MM treated with BTZ with or without DEX had indicated a median time to progression of 6.22 months in the APEX study,^20^ and 6.6 months in the SUMMIT study.^21^ We therefore decided that 6 months would be appropriate for stratifying patients in terms of PFS, which was defined as the period between initiation of BD therapy and either when progression was confirmed based on the International Myeloma Working Group uniform response criteria or when the patient died of any cause. As shown in Table 1, there were no significant differences of age or sex between the two groups. Regarding prior treatment, the group with shorter PFS tended to be more heavily treated and more often pretreated with immunomodulatory agents than the group with longer PFS. In terms of the cytogenetic risk category, the group with longer PFS included more patients with high-risk features (*n*=12/22, 48.0%) compared with the group with short PFS (*n*=10/34, 30.3%). The best overall response to BD therapy, that is, PR or better, was more frequent in the longer PFS group than the short PFS (100% vs 48.4%).

### Expression of the two ATF family members, ATF3 and ATF4, is significantly lower in primary MM cells from patients with shorter PFS

**Figure 1:**
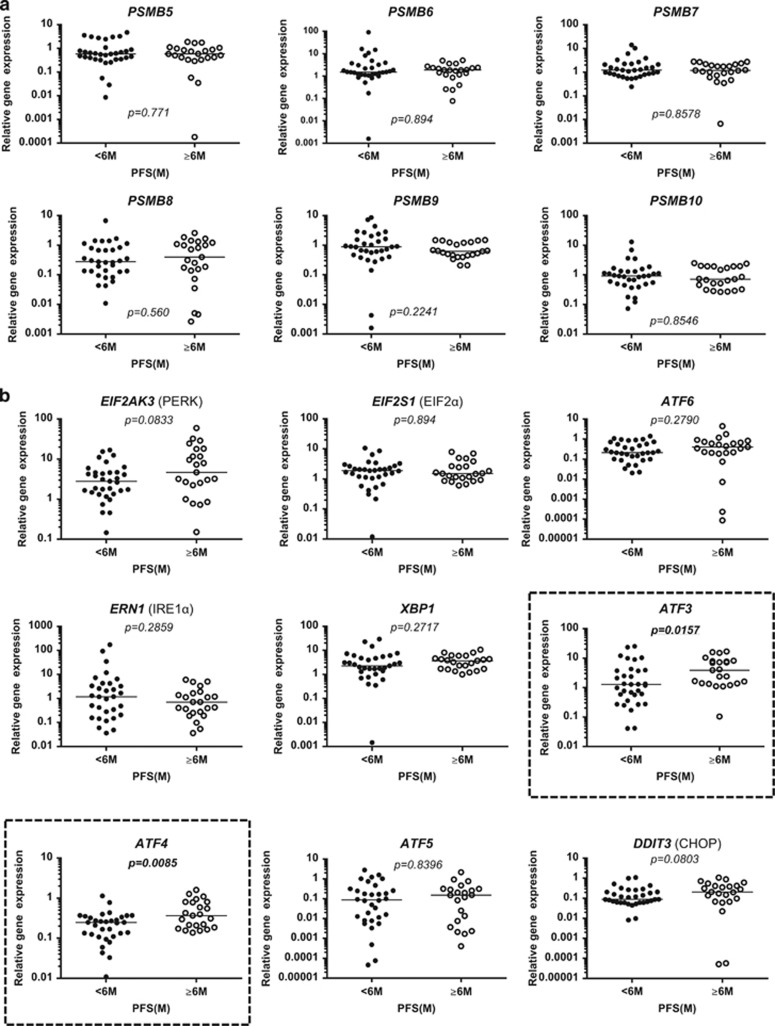
Basal mRNA expression of genes associated with proteasome and ER stress-related pathways in primary myeloma cells from patients receiving combination BTZ and DEX therapy. (**a**) Comparison of mRNA levels of six proteasome-related genes in patients with shorter PFS (<6 months, *n*=33) or longer PFS (⩾6 months, *n*=23). (**b**) Comparison of mRNA levels of nine unfolded protein response genes.

We quantified the amounts of mRNA for six proteasome, five UPR and four ER stress-related genes in purified MM cells. There were no significant differences for the six proteasome genes between the two groups (Figure 1a). Similarly, the expression of five UPR genes (*EIF2AK3*, *EIF2S1*, *ATF6*, *ERN1* and *XBP1*) was also found not to be significantly different. However, among the four genes associated with ER stress-related apoptosis, *ATF3* and *ATF4* were significantly more weakly expressed in MM cells from patients with a shorter PFS. Thus median values for *ATF3* mRNA were 1.3 vs 4.0, *P*=0.0157 and for *ATF4* mRNA 0.25 vs 0.38, *P*=0.0085 in the shorter and longer PFS groups, respectively (Figure 1a). To confirm the minimal possibility of influence from genomic DNA contamination during the evaluation of the expression levels of *ATF* genes, we prepared two RNA samples, with or without DNase treatment, from each of the three MM cell lines, KMS-11, U266 and RPMI-8226. Then we compared the expression levels of *ATF* mRNA between two samples. As shown in Supplementary Figure, the ratio of *ATF* expression in DNase I-treated to non-treated RNA was almost equal, suggesting that no significant difference existed in the *ATF* expression values between the DNase I-treated and non-treated samples.

Next we compared the expression of the same 15 genes between BD non-responders and responders, defining the former as patients with stable or progressive disease, and the latter as those achieving PR or better. However, there were no significant differences in the levels of expression of these genes between the two groups (data not shown).

### Lower basal mRNA expression of either or both ATF3 and ATF4 is associated with shorter PFS after BD therapy

**Figure 2:**
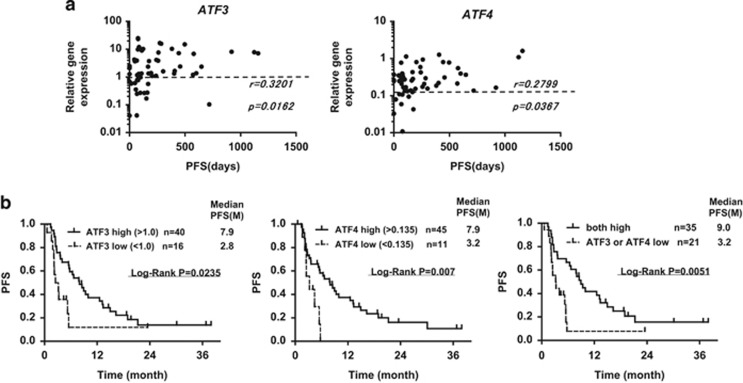
Relationship between the expression of *ATF3* and *ATF4* and PFS. (**a**) Correlation between PFS and *ATF3* or *ATF4* (Spearman's non-parametric *R*). (**b**) Kaplan–Meier curves for PFS, according to the expression of *ATF3* or *ATF4*. Cutoff values of *ATF3* and *ATF4* were 1.0 and 0.134, respectively. Left-hand panel: comparison of PFS between *ATF3* low and high groups; middle panel: *ATF4* low vs high; right-hand panel: both *ATF3* and *ATF4* high vs either *ATF* low.

Next we determined correlations between the expression of *ATF3* or *ATF4* and PFS after starting BD therapy in all 56 patients. As shown in Figure 2a, expression of either *ATF* gene correlated positively with PFS, whereas the other 10 genes tested showed no correlation (data not shown). To identify patients with the shortest PFS, we generated receiver operating characteristic curves and found that the cutoff values for ATF3 and ATF4, established as 1.0 and 0.134, respectively, were suitable for identification of a short PFS group with high predictability and specificity (Figure 2a, Table 2). The PFS is significantly shorter in patients with *ATF3* levels of ⩽1.0 relative to those >1.0 (median PFS: 2.8 vs 7.9 months; Figure 2b, left). Patients with *ATF4* levels of ⩽0.134 had shorter PFS than those with higher amounts (median PFS: 3.2 vs 7.9 months; Figure 2b, middle). Moreover, MM patients harboring low expression of either *ATF3* or *ATF4* mRNA had less favorable outcomes in terms of PFS than those with higher levels of both ATF3 and ATF4 (median PFS: 3.2 vs 9.0 months; Figure 2b, right).

### Prediction of shorter PFS under BD therapy by evaluation of ATF gene expression in MM cells

Importantly, most patients with low expression of *ATF* genes had shorter PFS under BD therapy, yielding a high positive prediction value of 95.2% (Table 2). Sensitivity and specificity of prediction of shorter PFS was 60.6% and 95.7%, respectively.

### BTZ treatment upregulates the expression of ATF3 and ATF4, followed by activation of ER stress-associated apoptosis in MM cells

**Figure 3:**
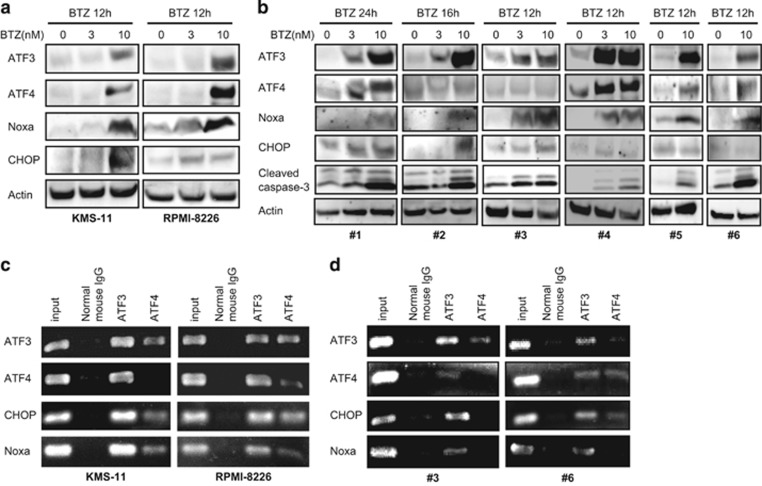
BTZ induces Noxa- and CHOP-dependent apoptosis via upregulation of *ATF3* and *ATF4*. (**a**, **b**) Two MM cell lines, KMS-11 and RPMI-8226, and six primary myeloma samples from patients were treated with different concentrations of BTZ and immunoblotted. (**c, d**) Two MM cell lines and primary myeloma samples #3 and #6 were treated with BTZ at 10 nm and subjected to chromatin immunoprecipitation with the indicated antibodies, followed by PCR amplification using specific primer pairs corresponding to promoter sequences of *ATF3, ATF4*, *CHOP* and *Noxa* genes.

Upon exposure to BTZ, expression of ATF3 and ATF4 was induced in two MM cell lines, KMS-11 and RPMI-8226. This was followed by activation of the pro-apoptotic signaling molecules CHOP and Noxa in a dose-dependent manner, resulting in caspase-dependent apoptosis through excessive ER stress (Figure 3a). The same phenomenon was similarly observed in primary MM cells from patients treated with BTZ (Figure 3b). All six samples tested showed upregulation of ATF3, Noxa and CHOP expression, four (#1, #4, #5, and #6) also concomitant ATF4 upregulation and another four (#1, #2, #3 and #4) upregulated CHOP. To examine the binding activity of the two ATF family members to promoter regions of the four genes *ATF3*, *ATF4*, *CHOP* and *Noxa*, the two MM cell lines were cultured in the presence of 10 nm BTZ and analyzed by ChIP. As shown in Figure 3c, both ATF3 and ATF4 bound to the promoter sequences of ATF3, CHOP and Noxa. ATF3 bound to the promoter sequences of ATF4 in both MM cell lines, while ATF4 bound itself only in RPMI-8226 cells (Figure 3c). There were sufficient primary MM cells for ChIP analysis from two patients, #3 and #6. As shown in Figure 3d, ATF3 bound to the promoter sequences of all four genes, while ATF4 bound only to the ATF3 promoter in case #3 and to ATF4 and CHOP promoter sequences in case #6.

### Silencing expression of ATF3 decreases BTZ-induced apoptosis via suppression of Noxa and DR5 induction

**Figure 4:**
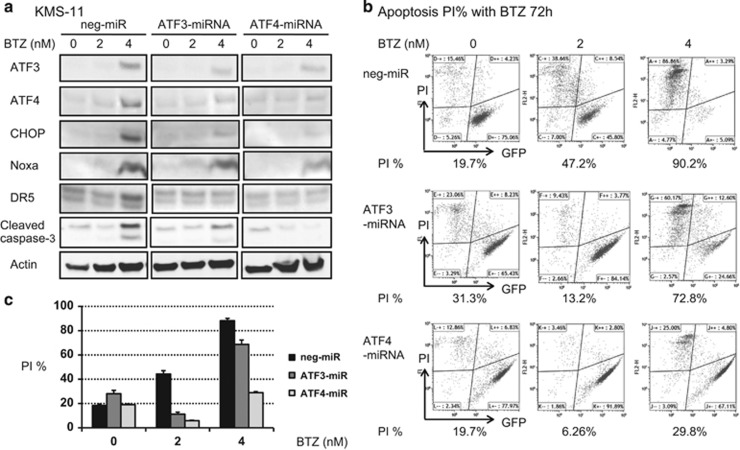
Stable knockdown of ATF3 and ATF4 expression by lentiviral miRNA. (**a**) Immunoblot analysis of miRNA-transfected KMS-11 cells. Altered expression of ATF3, ATF4, CHOP, Noxa and caspase 3 was analyzed before and after BTZ treatment. (**b, c**) Comparison of BTZ-induced apoptosis in miRNA-transfected cells. Apoptosis was assessed by measuring cells stained by PI.

Two stable clones derived from KMS-11 cells with suppressed basal expression levels of either ATF3 or ATF4 were established by infection with two different lentiviruses, each carrying a miRNA sequence targeting ATF3 or ATF4. When either ATF3 or ATF4 was silenced, amounts of other ATF, pro-apoptotic factors CHOP, Noxa, and DR5 were decreased and caspase activation entirely suppressed during BTZ treatment. KMS-11 cells with either ATF3 or ATF4 knocked down showed less apoptotic progression than mRNA-transfected negative controls, as reflected in a low frequency of PI-positive cells (Figure 4b). The percentage of PI-positive cells in negative controls, ATF3 knocked down cells and ATF4 knocked down cells was 47.2, 13.2 and 6.3% at a 2 nm concentration of BTZ and 90.2, 72.8 and 29.8% at a 4 nm concentration of BTZ, respectively (Figures 4b and c).

### ATF families execute ER stress-induced apoptosis on BTZ treatment of MM cells

**Figure 5:**
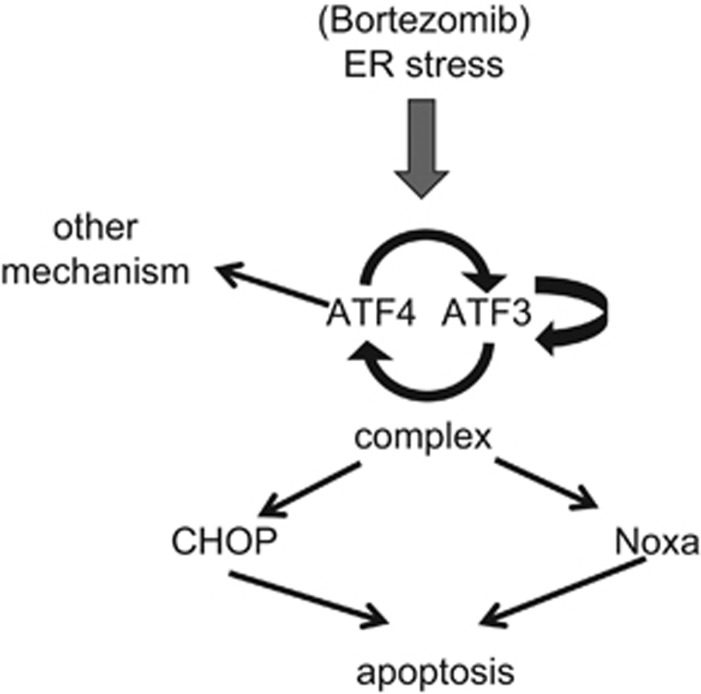
Scheme of the role of *ATF* genes during ER stress-induced apoptosis. Summary of ER stress-induced apoptosis triggered by proteasome inhibition in myeloma cells, focusing on the role of ATF family members during prolonged ER stress.

Our current understanding of ER stress-induced apoptosis caused by proteasome inhibition in MM cells is summarized in Figure 5, based on our present study and earlier reports. When proteasome function is inhibited, ER overload occurs, leading to fatal ER stress, and this transactivates several transcription factors, including the two ATFs, ATF3 and ATF4. Their overexpression then leads to transactivation of other *ATF* target genes, and upregulation of ATF3 can lead to its self-activation. Moreover, ATF3 and ATF4 form heterodimeric complexes, which subsequently result in transactivation of the pro-apoptotic factors CHOP and Noxa, the final executers of ER stress-induced apoptosis.

## Discussion

In the current study, we observed lower basal expression of two *ATF* mRNAs in patients with relapsed/refractory MM who had relatively short PFS under BD therapy. The mechanism of action of BD on MM cells would be expected to be highly dependent on the ER stress inducing apoptosis through the activation of ATF3 and ATF4 during BTZ treatment.

Regarding the cytogenetic risk category of the MM cells, counter-intuitively, the group with longer PFS included more patients with high-risk features. The reason for this is unclear, but it may be that, following the introduction of proteasome inhibitor and immunomodulatory drugs, MM cases with the t(4;14) karyotype are no longer always to be considered clinically high risk. They may be considered to be a more heterogeneous group. Therefore, we propose that new biomarkers, such as ATF family members, may contribute to stratifying this heterogeneous group in terms of sensitivity to BTZ therapy.

Suppression of *ATF* gene expression may contribute to the suboptimal efficacy of BTZ-containing therapy in some MM patients. This idea is supported by the fact that low expression of the two ATF significantly reduced BTZ-induced apoptosis via inhibited induction of the pro-apoptotic factors Noxa and CHOP. This finding is partly consistent with a previous report that sensitivity to BTZ was associated with the expression of several *ATF* genes, including ATF3, ATF4 and ATF5, in BTZ-sensitive or -resistant non-Hodgkin's B-cell lymphoma-derived cell lines.^22^ Higher basal expression of ATF family members may reflect cell exposure to strong ER stress. Hence, BTZ inhibition of the proteasome changes the fate of the cells to undergo apoptosis.

To date, there have been few studies reporting specific biomarkers for predicting the efficacy of BD therapy in MM patients. These include three genes, *XBP1*,^19, 23, 24, 25, 26^ *KLF9*^25^ and *Nampt*,^27^ which are associated with UPR, a trans-activator of Noxa and a key enzyme involved in NAD^+^ metabolism, respectively. Of these three, XBP1 is involved in UPR components, and the IRE1α-XBP1 pathway has been implicated in the proliferation and survival of MM cells.^28, 29^ In our study, however, we did not find any correlation of expression of genes in this pathway with the duration of PFS in patients under BD therapy. Among other possibilities, this may be for at least two reasons. First, unlike in our study, the previous report adopted the result of the best response to BD therapy, rather than the duration of response or PFS, when evaluating the correlation of clinical efficacy with *XBP1* expression. Our study included several patients who showed suboptimal responses to BD therapy, such as stable disease or minimal response, which nonetheless resulted in a long duration of PFS. Second, another reason may be that evaluation of XBP1 mRNA expression was not performed for each isoform, such as spliced and un-spliced forms in our study. Because a splice position in *XBP1* mRNA is located 1 kb away from the 3′ end of the transcript,^30, 31^ there was no suitable specific probe to identify splice variants of all isoforms when using amplified RNA samples. It is possible that expression of the active form, that is, a spliced *XBP1* isoform, may be associated with the efficacy of BTZ.

In previous reports regarding the mechanism of action of BTZ-induced cancer cell apoptosis, accumulation of misfolded proteins followed by fatal ER stress,^16, 32^ inactivation of the nuclear factor-κB pathway,^33^ mitochondrial membrane injury facilitated by BH3-only proteins, including Noxa, Bid, puma and Bik, and cleaved Mcl-1 isoform, all seemed indispensable.^13, 34, 35, 36^ However, little was known about how prolonged ER stress activates these pro-apoptotic factors. Using mouse embryonic fibroblast cells treated with BTZ or ERAD inhibitors, Wang *et al.*^37^ were the first to demonstrate that prolonged ER stress ultimately transactivates ATF3 and ATF4 expression and promotes the formation of ATF family complexes, which directly transactivate the pro-apoptotic factors CHOP and Noxa. Consistent with their report, our study using MM cells also demonstrated that BTZ-induced expression of ATF3 and ATF4 regulates the expression of CHOP and Noxa through binding to their promoter sequences, resulting in transactivation of these pro-apoptotic factors. According to our data, silencing ATF4 expression impairs BTZ-induced apoptosis via the reduced expression of CHOP and Noxa more markedly than silencing ATF3 expression. The reason for this remains unknown. It may be speculated that, in addition to forming complexes with ATF3, ATF4 also forms complexes with other transcription factors, such as other ATF family members, and transactivates ER stress-associated pro-apoptotic factors in an ATF3-independent manner.^38^ In addition, ATF4 was recently implicated in regulating autophagy through the modulation of Atg5 and Atg12 expression during prolonged ER stress, considered to be a major cell death pathway in ER stress-induced apoptosis.^39^ To address the question of ATF3-independent cell death mechanisms, we are currently determining which other transcription factors form complexes with ATF4 and the mechanisms responsible for the influence of ATF4 on autophagy in BTZ-treated MM cells, including primary samples.

In summary, we have shown that low expression of *ATF* genes is associated with suboptimal efficacy of BTZ treatment. We have documented that ATF acts as a novel and potentially clinically relevant transcriptional regulator in BTZ-induced apoptosis of MM cells. Although the amounts of *ATF3* and *ATF4* mRNA in MM cells before treatment could be potential predictive biomarkers of the efficacy of BD therapy or of other BTZ-containing regimens, clinical utility needs to be confirmed by large-scale replication studies in patients with MM. Our findings contribute to a better understanding of the roles of ATFs in ER stress-related apoptosis and also suggest that ER stress-associated pathways may be potential new options for molecular targeting therapy in MM.

## Figures and Tables

**Table 1:** Patient demographics and baseline characteristics

*Characteristic*	*No. of patients*	
	*PFS<6 months*	*PFS*⩾*6 months*
Total number	33	23
		
*Sex*		
Men	15 (45.4%)	12 (52.2%)
Women	18 (54.5%)	11 (47.8%)
		
Age: median±s.d., years	64±11.3	67±10.2
		
*Prior treatment:*		
⩾3	11 (33.3%)	3 (13.0%)
2	11 (33.3%)	5 (21.7%)
1	7 (21.2%)	13 (56.5%)
0	4 (12.1%)	2 (8.7%)
		
*IMID history*	15 (45.4%)	4 (26%)
Thalidomide	11	3
Lenalidomide	4	3
		
*High-risk cytogenetic or molecular genetic aberrations*	10 (30.3%)	12 (52.0%)
t(4;14) or FGFR3(+)	5	10
t(14;16) or MAFB(+)	3	1
del(13q) or del(17p)	2	1
		
*Best response*^a^*: PR over*	16 (48.4%)	23 (100%)
CR+VGPR	6	10
PR	10	13
SD	10	0
PD	5	0
UNK	2	0

Abbreviations: CR, complete response; IMID, immunomodulatory drug; PD, progressive disease; PFS, progression-free survival; PR, partial response; SD, stable disease; VGPR, very good partial response; UNK, unknown.

a According to the International Uniform Response Criteria.

**Table 2:** Prediction of poor response to BD treatment according to basal expression of ATF3 and ATF4 in primary myeloma cells

*Expression values of ATF3 and ATF4 genes*	*PFS (months)*		*Total*	*Prediction (%)*
	*<6*	⩾*6*		
Either low (ATF3<1.0 or ATF4<0.135)	20	1	21	95.2 (positive predictive value)
Both high	13	22	35	62.9 (negative predictive value)
Total	33	23	56	
	60.6 (sensitivity)	95.7 (specifity)		

Abbreviations: ATF, activating transcription factor; BD, bortezomib+dexamethasone; PFS, progression-free survival.
