# Supplementary material for: Lower expression of activating transcription factors 3 and 4 correlates with shorter progression-free survival in multiple myeloma patients receiving bortezomib plus dexamethasone therapy
Source: Blood Cancer J. 2015 Dec 4;5(12):e373–. doi: 10.1038/bcj.2015.98 (PMC4735074; doi:10.1038/bcj.2015.98)
Supplement: Supplementary Figure [file bcj201598x1.ppt]

## Slide 1
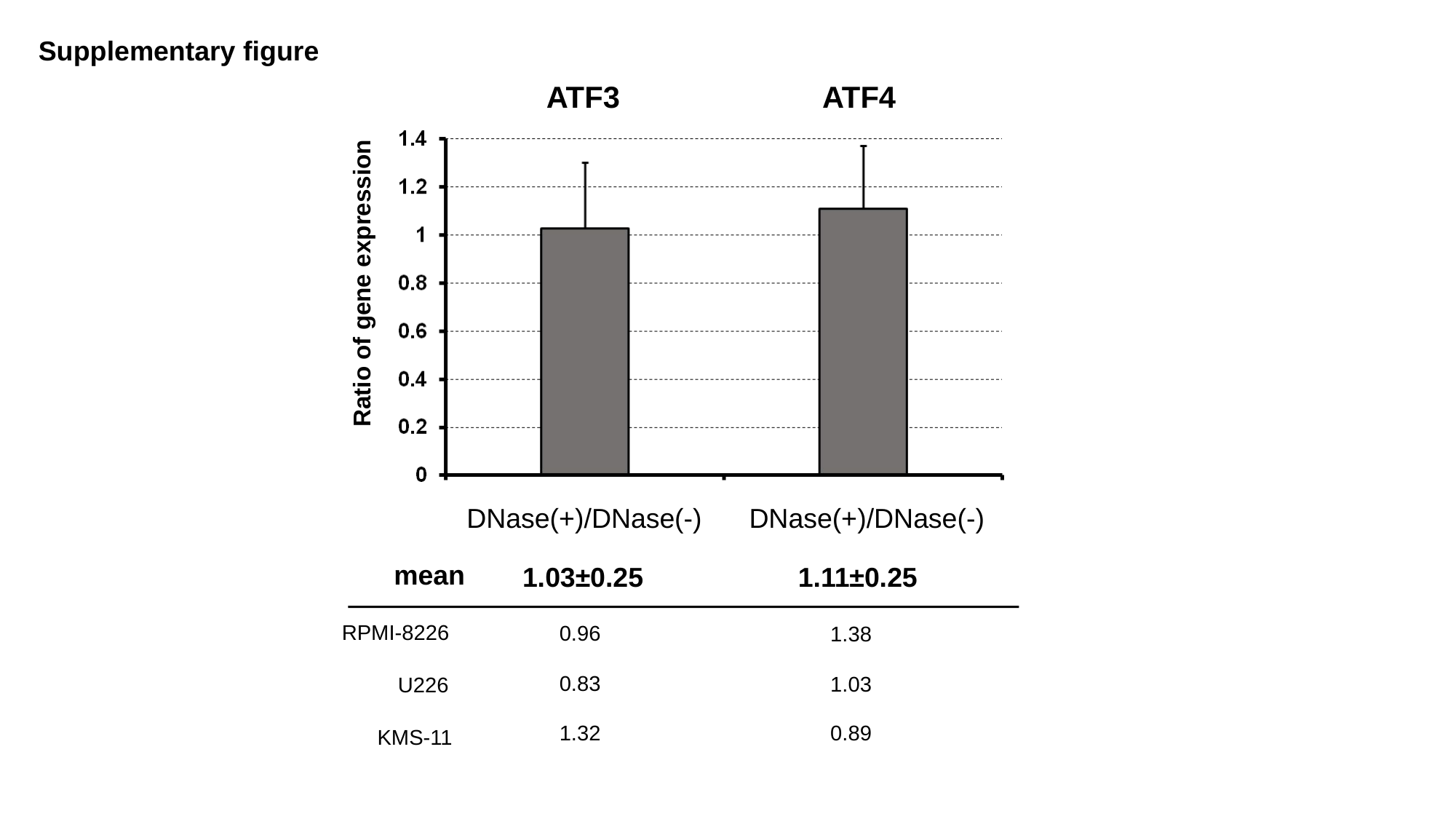

Supplementary figure
ATF3
ATF4
Ratio of gene expression
DNase(+)/DNase(-)
DNase(+)/DNase(-)
mean
1.03±0.25
1.11±0.25
RPMI-8226
0.96
1.38
0.83
1.03
U226
1.32
0.89
KMS-11
