# Supplementary material for: Lower expression of activating transcription factors 3 and 4 correlates with shorter progression-free survival in multiple myeloma patients receiving bortezomib plus dexamethasone therapy
Source: Blood Cancer J. 2015 Dec 4;5(12):e373–. doi: 10.1038/bcj.2015.98 (PMC4735074; doi:10.1038/bcj.2015.98)
Supplement: Supplementary Figure Legend [file bcj201598x2.doc]

**Supplementary Figure legends**

**Comparing *ATF3* and *ATF4* gene expression in DNase-treated or non-treated RNA samples from three multiple myeloma cell lines**

*ATF* mRNA expression was measured in RNA samples, and mean *ATF3* or *ATF4* mRNA expression ratio in DNase-treated to non-treated RNAs in three cell lines was evaluated.
